# PGC7 maintains the pluripotency of F9 embryonic carcinoma cells by promoting Nanog translation: PGC7 promotes Nanog translation

**DOI:** 10.3724/abbs.2025035

**Published:** 2025-03-11

**Authors:** Yingxiang Liu, Xing Wei, Caixia Zhang, Jingya Liu, Mengying Yu, Peiwen Feng, Zekun Guo

**Affiliations:** 1 Orthopedic Oncology Institute Department of Orthopedic Surgery Tangdu Hospital Fourth Military Medical University Xi’an 710000 China; 2College of Veterinary Medicine Northwest A&F University Yangling 712100 China; 3 Human Phenome Institute Fudan University Shanghai 200433 China; 4 Xi’an Center for Disease Control and Prevention Xi’an 710049 China; 5 College of Life Science Northwest A&F University Yangling 712100 China

**Keywords:** primordial germ cell 7 (PGC7), Y-box binding protein 1 (YBX1), Nanog, pluripotency

## Abstract

Primordial germ cell 7 (PGC7) is prominently expressed in primordial germ cells (PGCs) and embryonic stem cells (ESCs), serving as a pivotal marker for discerning stem cell pluripotency. However, the role of PGC7 in regulating core pluripotency factors remains unclear. In this study, the expression dynamics of PGC7 and pluripotency-associated proteins are systematically evaluated by quantitative reverse transcription PCR (RT-qPCR) and western blot analysis. Complementary experimental approaches including confocal immunofluorescence and Co-immunoprecipitation (Co-IP) assays are subsequently employed to establish subcellular colocalization patterns and elucidate the molecular mechanisms associated with PGC7 function. The results show that PGC7 is closely associated with the pluripotency status of F9 embryonal carcinoma (EC) cells. Notably, PGC7 can counteract the decrease in pluripotency induced by retinoic acid (RA). Ectopic expression of PGC7 in F9 EC cells enhances the translation of Nanog. Mechanistic analysis reveal that PGC7 activates Y-box binding protein 1 (YBX1) phosphorylation by enhancing the interaction between YBX1 and AKT1. The subsequent phosphorylation of YBX1 reduces its binding to Nanog mRNA and promotes the translation of Nanog. These results shed light on a previously unknown role of PGC7 in supporting the translation of Nanog, offering valuable insights into the functions of PGC7 in F9 EC cells.

## Introduction

With their potential for unlimited proliferation, pluripotency and low immunogenicity, embryonic stem cells (ESCs) have become crucial tools in the fields of regenerative medicine and disease treatment [ 1, 2]. Primordial germ cell 7 ( *PGC7*) is an essential maternal effect gene in early embryonic development and is specifically expressed in germ cells, oocytes, preimplantation embryos, and pluripotent cells [3]. The differential expression and functional diversity of PGC7 in these distinct cell types are crucial for comprehending its role in developmental biology [ 4, 5]. PGC7 is a small, basic protein with a molecular weight of approximately 17 kDa that is characterized by a putative SAP-like domain and a splicing-like factor [6]. Current research on PGC7 has focused on its ability to regulate DNA methylation. In NIH-3T3 cells, the interaction between PGC7 and UHRF1 inhibits the recruitment of DNMT1, leading to genome-wide DNA hypomethylation [7]. In F9 embryonal carcinoma (EC) cells, PGC7 also regulates ERK-mediated DNMT1-Ser717 phosphorylation, affecting genome-wide methylation [8]. Furthermore, PGC7 promotes DNA demethylation by directly binding to the plant homeodomain of UHRF1, disrupting the association of UHRF1 with chromatin [9]. Additionally, PGC7 counteracts DNMT3A-mediated demethylation through its interaction with HP1BP3 [10]. During the maturation of oocytes, the absence of PGC7 results in abnormal accumulation of UHRF1 and DNMT1 in the nucleus. This ultimately causes excessive DNA methylation across the genome, including the promoters of inactive genes, and further impairs zygotic genome activation (ZGA) [11]. PGC7 must localize to the nucleus to protect the maternal genome from demethylation, a process initiated by the interaction between PGC7 and Ran-binding protein 5 (RBP5). Further studies revealed that PGC7 primarily binds to dimethylated histone H3 lysine 9 (H3K9me2), thereby inhibiting the activity of Tet3 methylcytosine oxidase in the maternal genome and at specific imprinted loci in the paternal genome [ 12– 14]. Knocking out *PGC7* results in widespread transcriptional dysregulation, which is characterized primarily by impaired activation of endogenous retroviruses (ERVs), ultimately leading to the failure of ZGA [15]. Although PGC7 is unique to mammals, it is also capable of inducing global DNA demethylation in non-mammalian species, such as Xenopus and medaka [16].

In both ESCs and germ cells, the expression of PGC7 serves as an indicator of pluripotency. PGC7 has heterogeneous expression patterns within ESCs. PGC7-positive ESCs resemble the inner cell mass (ICM), whereas PGC7-negative cells resemble epiblast cells and exhibit a greater propensity for neural and trophectoderm differentiation [17]. PGC7 is exclusively expressed in low-grade (lg) and high-grade (hg)-induced pluripotent stem cells (iPSCs). The addition of exogenous PGC7 significantly improves the efficiency of reprogramming, leading to a greater yield of high-grade iPSCs. This enhancement is achieved by maintaining the imprinting of the IG-DMR at the Dlk1-Dio3 locus through the suppression of DNMT3A enrichment [18].

PGC7 is an intrinsically disordered protein IDP, and its function primarily depends on its interacting partners [19]. Our previous study revealed that PGC7 interacts with Y-box binding protein 1 (YBX1) [20]. YBX1 is a well-known, highly expressed RNA-binding protein (RBP) that serves as a critical regulator of transcription and translation within cells. YBX1 binds to DNA or RNA through an evolutionarily conserved cold shock domain (CSD), while the C-terminal domain (CTD) of YBX1 contains alternating basic and acidic clusters, which is implicated in protein-protein interactions [ 21, 22]. In the cytoplasm, YBX1 is involved primarily in mRNA-dependent processes, including mRNA translation and pre-mRNA splicing [ 23, 24]. The consensus binding site in mRNAs targeted by YBX1 is known as the CU-box; YBX1 binding not only protects its target mRNAs from degradation but also inhibits their translation by antagonizing eIF4G [ 25– 28]. YBX1 plays a pivotal role in regulating epithelial-mesenchymal transition (EMT) by promoting the cap-dependent translation of mRNAs that encode factors facilitating EMT [29]. Additionally, YBX1 negatively regulates the senescence of epidermal progenitors by controlling the translation of specific cytokine mRNAs associated with senescence, which are regulated through their 3′ untranslated regions (3′UTR) [30]. In adult progenitor cells, YBX1 partners with DDX6 to target stem loops within the 3′UTRs of key regulators of proliferation and self-renewal, such as CDK1 and EZH2, and recruits them to eIF4G to increase their translation [31]. In zebrafish, YBX1 governs Nodal signaling by binding to the 3′UTR of sqt RNA, thereby inhibiting its premature translation [32]. YBX1 is involved in the regulation of cellular pluripotency. In adenocarcinoma stem cells, the interaction between YBX1 and ERα controls the stemness and differentiation of ER-positive breast cancer stem cells [33]. During the osteogenic differentiation of MSCs, the expression of YBX1 gradually increases. *YBX1* knockdown can negatively regulate the osteogenic differentiation of MSCs [34].

The function of YBX1 is regulated primarily by its phosphorylation at various sites by multiple kinases. Mass spectrometric studies of the phosphoproteome revealed that YBX1 is phosphorylated at several amino acid residues, including Ser102, Ser165 and/or Ser167 [35]. The phosphorylation induced by the kinases ERK2 and GSK3β enhances the binding of YBX1 to the VEGF gene promoter [22]. Additionally, AURKA phosphorylates YBX1 at two key residues, T62 and S102, which stabilizes YBX1 and promotes its nuclear translocation [36]. Phosphorylation at Ser102 (equivalent to serine 100 in mice) by AKT1 plays a vital role in activating translation. Phosphorylated YBX1 dissociates from the capped 5′UTR of mRNAs, thereby increasing the affinity of eIF4G for the 5′UTR of mRNAs [37]. The function of YBX1 can be regulated by its interacting proteins [38]. The interaction between YBX1 and C1QBP is essential for YBX1 phosphorylation, and knockdown of *C1QBP* enhances the phosphorylation of YBX1 and its nuclear translocation.

The homeoprotein Nanog is specifically expressed in undifferentiated embryonic stem cells, the inner cell mass (ICM) of early embryonic development, and pluripotent cells, and plays important functions in these stages, such as maintaining the pluripotency and self-renewal ability of ESCs [39]. Elevated levels of Nanog can sustain mouse self-renewal independently of leukemia inhibitory factor (LIF) and facilitate human ESCs growth without the need for feeder cells. ESCs deficient in Nanog lose their pluripotency and differentiate into the extraembryonic endoderm lineage [40]. Two neighboring super-enhancers are located between *Pgc7* and *Nanog*, with –45 eRNAs produced at the –45SE enhancer that specifically regulate *Pgc7* by stabilizing chromatin looping [41]. PGC7 is expressed earlier than Nanog, from the embryonic stage to the ICM period. Therefore, PGC7 is a key factor in regulating post-implantation embryonic pluripotency. The regulation of Nanog at the post-transcriptional level, particularly in terms of translational regulation, is less well understood. The PGC7-interacting proteins in ESCs are primarily involved in translation [42]. However, specific studies investigating how PGC7 regulates gene expression at the translational level are lacking.

In this study, we demonstrated that PGC7 is closely associated with the pluripotency status of F9 EC cells. PGC7 antagonizes the RA-induced differentiation of F9 EC cells, primarily by sustaining the translation of Nanog. PGC7 interacts with YBX1, co-localizing in the cytoplasm, and activates translation by enhancing YBX1-Ser100 phosphorylation through a strengthened interaction with AKT1. In summary, we explored the translational regulatory mechanism of PGC7 and its interacting protein YBX1 on the pluripotency factor Nanog.

## Materials and Methods

### Cell culture

Mouse F9 EC cells and HEK-293T cells were obtained from the Cell Bank of the Chinese Academy of Sciences (Shanghai, China). The cells were cultured in Dulbecco’s modified Eagle’s medium (Pricella, Wuhan, China) supplemented with 10% fetal bovine serum (Invitrogen, Carlsbad, USA). Cell culture was performed as previously described [43]. Upon reaching 80% confluence, the cells were passaged. In accordance with the requirements of subsequent experiments, the cell suspension was slowly added to culture dishes or plates in proportion, gently shaken to ensure even distribution, and then transferred to an incubator for continued culture. All the cell lines used in this study were maintained in a humidified 5% CO _2_ incubator at 37°C, with culture medium supplemented with 100 U/mL penicillin and 100 μg/mL streptomycin to ensure optimal growth conditions.

### Construction of plasmids

The coding sequences of the *Pgc7*, *Akt1* and *Ybx1* genes were amplified from F9 EC cells and then cloned and inserted into the pCMV-HA, pEGFP-C1 or p3×Flag-CMV-10 vector (preserved in our lab). The sequences of the primers used are listed in Supplementary Table S1. The construction of the vector was carried out according to the standard protocol.

### Cell transfection

Short interfering RNAs (siRNAs) targeting mouse *Pgc7* were purchased from GenePharma (Shanghai, China), and the sequences used are detailed in Supplementary Table S2. Upon reaching 50%–60% confluence, the cells were prepared for transfection. siRNAs and Lipofectamine 2000 (Life Technologies, Carlsbad, USA) were each mixed with Opti-DMEM (11058021; Gibco, Waltham, USA) and incubated for 5 min. The two mixtures were then combined and incubated at room temperature for 20 min. The resulting mixture was added to the cell culture dishes, gently swirled to ensure even distribution, and incubated at 37°C for 6 h. The medium was subsequently replaced by fresh culture medium, and the cells were allowed to grow for an additional 24–48 h. For the PGC7, YBX1 or AKT1 overexpression experiment, media were changed ~6 h post-transfection and the cells were harvested ~48 h post-transfection.

### Reverse transcription PCR and real-time quantitative PCR (RT-qPCR) analysis

Total RNA extraction, reverse transcription, and RNA quantification were performed according to standard methods. Reverse transcription was performed via the PrimeScript RT Kit (Takara, Dalian, China). The fluorescent quantitative PCR system was configured using the TB Green ^®^ Premix Ex Taq™ II (Tli RNaseH Plus; Takara). The cycling program was as follows: an initial denaturation step at 95°C for 30 s, followed by 40 cycles of denaturation at 95°C (5 s), annealing at 60°C (30 s), and extension at 72°C (30 s). For detailed experimental procedures, please refer to our previous study [44]. *Gapdh* was used as a control to normalize the expression of the test genes. The 2 ^–ΔΔCT^ method was used to analyze relative changes in the expression of genes. All sequences of primers used for qPCR are shown in Supplementary Table S3.

### Alkaline phosphatase (AP) staining

F9 EC cells were passaged into 6-well plates and treated according to different experimental requirements when the cell density reached 60%. After 24–48 h, AP staining was performed via an AP staining kit (Beyotime, Nantong, China) following the manufacturer’s instructions. The cells were fixed with 4% paraformaldehyde for 5 min after washing with PBS and incubated with AP solution (BCIP and NBT) at room temperature for 10–15 min. PBS was then added to terminate the reaction then washed three times with PBS. The staining intensity of the AP was measured via Image J software.

### Co-immunoprecipitation

Here we take the interaction between Flag-PGC7 and HA-YBX1 as an example to describe the method. After 48 h of transfection, HEK-293T cells were washed twice with precooled PBS and lysed in ice-cold IP lysis buffer (Pierce, Rockford, USA) with proteinase inhibitor cocktail (26146; Thermo Fisher Scientific, Waltham, USA) on ice for 30 min. The cell lysate was incubated with the anti-Flag antibody (Thermo Fisher Scientific) at 4°C overnight. Mouse IgG1 Isotype control (Thermo Fisher Scientific) was used as a negative control. Next, 0.2 mg of Protein A/G Magnetic Beads (Pierce) were added to each sample and incubated at 4°C for 2 h. Then the pellets were collected, washed with IP wash buffer (Pierce), and bound proteins were eluted with 1× SDS sample buffer by boiling at 100°C for 10 min. The methods used were the same as those described in our earlier papers [20]. The immunoprecipitates were subsequently analyzed via western blot analysis. The protein molecular mass encoded by pCMV-HA-YBX1 could be approximately 50 kDa. To avoid overlapping signals of the IgG heavy chain or light chain, an HRP-conjugated light-chain-specific or heavy-chain-specific secondary antibody (Abbkine, Wuhan, China) was used.

### Confocal microscopy

Briefly, F9 EC cells were seeded onto glass coverslips and harvested at 36 h post-transfection with GFP-PGC7 plasmids. Cells were fixed with 4% paraformaldehyde at room temperature for 10 min. Cells were incubated with the YBX1 antibody overnight at 4°C, followed by 3 time of washes with washing buffer (Beyotime) for 5 min and then and labelled with corresponding Alexa Fluor‐546 conjugated secondary antibodies for 2 h at room temperature, followed by DAPI staining. For detailed experimental procedures, please refer to our previous research [10]. The antibodies used are shown in Supplementary Table S4. Confocal microscopy images were obtained via a TCS-SP5 confocal laser scanning microscope (Leica Microsystems, Heidelberg, Germany).

### Western blot (WB) analysis

The differently treated cells were lysed with ice-cold RIPA buffer (Beyotime) supplemented with protease inhibitors (Roche, Mannheim, Germany) on ice for 30 min. The BCA protein assay reagent (Pierce) was used to quantify the protein concentration of the lysates. The total protein samples were denatured, separated on 12% polyacrylamide gels and transferred to PVDF membranes (Millipore, Billerica, USA). After being blocked for 2 h at room temperature in 5% nonfat dry milk in TBST (10 mM Tris-HCl, 150 mM NaCl, and 0.1% Tween-20, pH 7.0), the membranes were then incubated with primary antibodies overnight at 4°C and then incubated with secondary antibody for 2 h at room temperature after washing three times with TBST for 10 min each. For detailed experimental procedures, please refer to our previous research [10]. All the antibodies used for WB analysis are shown in Supplementary Table S4. The immunoblots were visualized via autoradiography wioth a Gel Doc™ XR+ Gel documentation system (Bio-Rad, Hercules, USA).

### RNA-binding protein immunoprecipitation (RIP)

The interaction between YBX1 and its target mRNAs was validated by RNA immunoprecipitation. For RIP with YBX1 antibody, F9 EC cells were removed from 100-mm culture plates and washed with cold RNase free PBS. Cells were collected by centrifugation at 352 *g* at 4°C and the supernatant was discarded. Then 100 μL RIP lysis buffer was added to the cell pellet and incubated the lysate on ice for 5 min and subsequently stored at –80°C overnight. The subsequent experiments were performed according to the published literature [ 26, 45] and the instructions of the Magna RIP™ RNA-Binding Protein Immunoprecipitation Kit (Millipore). Immunoprecipitated RNAs and proteins were purified for analysis. Western blot analysis was used to verify the specificity of YBX1 antibody. RT-qPCR was applied to detect the RNAs enriched by YBX1. The amount of RNA coprecipitated with the antibody was calculated as a percentage of total input via the following formula: ΔCT = CT _input_ – CT _RNA IP_, %total = 2ΔCT × 10%. The signal from the input samples represented 10% of the total RNA used in each RIP. The percentage of total inputs above 0.1% was considered significant.

### Protein synthesis efficiency

HPG was detected using a Click-iT ® HPG Alexa Fluor ® Protein Synthesis Assay Kit (Life Technologies) according to the manufacturer’s instructions. Analyses were performed 36 h after si-RNA transfection. Before treatments, cells were cultured in a medium without methionine (Life Technologies) for 40 min to deplete endogenous methionine. The mean intensity of the HPG signal was measured across each field and quantified via ImageJ software. At least 6 fields for each group were counted to determine the mean intensity of the HPG signal.

### Treatment with inhibitors

In this study, cells grown in 6-well plates served as the experimental model. Treatment preparation commenced when the cells reached 50% to 60% confluence. The media from the culture plate were removed, and the cells were rinsed once with PBS before being provided with new medium containing either the inhibitors or DMSO. They were subsequently returned to the incubator for further incubation. Western blot analysis or AP assay was conducted after a treatment duration of 6–24 h, depending on the experimental objectives. Additional information regarding the inhibitors can be found in Supplementary Table S5.

### Sequence analysis

The sequence analysis of the consensus binding site in Nanog mRNA targeted by YBX1 was performed using MegAlign in DNAStar Lasergene V 7.10 (DNAstar, Madison, USA).

### Statistical analysis

Data are expressed as the mean ± standard deviation (SD). Differences between two groups were compared via a two-tailed paired Student’s *t* test, and significance was set at *P*  < 0.05. The error bars in the figures represent the SD of at least three biological replicates.

## Results

### PGC7 is tightly associated with the pluripotency status of F9 EC cells

**Figure 1:**
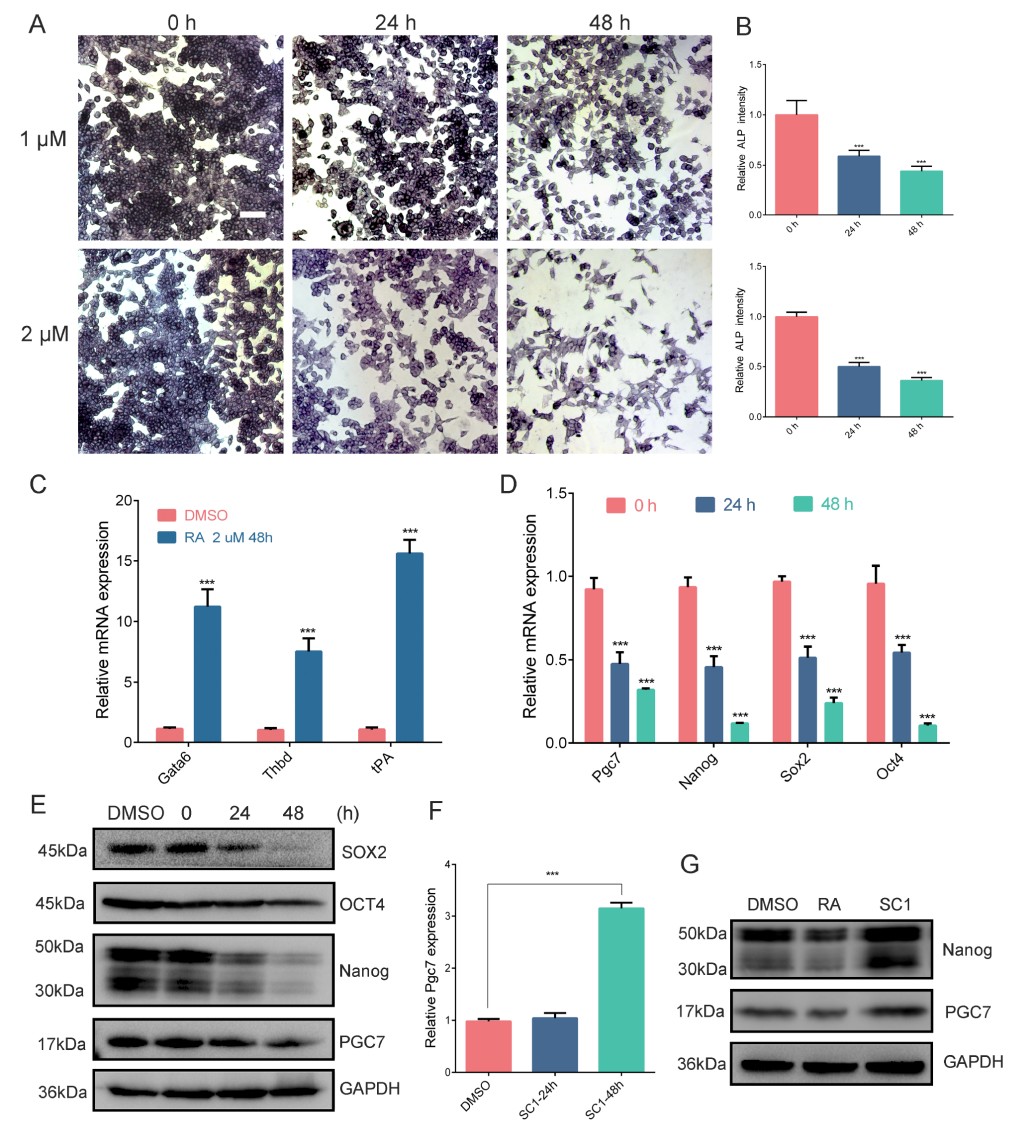
Figure 1 PGC7 is tightly associated with the pluripotency status of F9 EC cells (A) ALP staining of F9 EC cells treated with an equal volume of DMSO or 1 μM or 2 μM RA for 24 h or 48 h. Scale bar = 80 μm. (B) Image J software was used to quantify the intensity of alkaline phosphatase staining and compare samples. (C) During the RA-mediated differentiation of F9 EC cells, the differentiation-related gene expression levels of Gata6, Thbd and tPA were analyzed via RT-qPCR. Data are presented as the mean ± SD of three independent experiments. Gapdh was used for normalization. (D,E) During the RA-mediated differentiation of F9 EC cells, PGC7 expression decreases in parallel with the expression of the pluripotency genes Nanog, Oct4 and Sox2, as measured by RT-qPCR (D) and western blot analysis (E). (F) F9 EC cells were treated with 1 μM SC1 or an equal volume of DMSO for 24 h or 48 h, and the expression levels of PGC7 were analyzed by RT-qPCR. (G) Western blot analysis of PGC7 and Nanog protein levels after F9 EC cells were treated with 1 μM RA, 1 μM SC1 or an equal volume of DMSO for 48 h. Data are presented as the mean ± SD of three independent experiments. ***P < 0.001, according to Student’s t test.

To investigate the association between PGC7 expression and pluripotency status, F9 EC cells, which exhibit high levels of PGC7 expression, were utilized in this study. RA is known to induce differentiation in F9 EC cells [46]. We examined whether RA signaling influences PGC7 expression. Following RA exposure, the cells were stained for AP, a marker of pluripotency. A significant decrease in AP activity was observed with increasing incubation time and RA dosage (1 μM or 2 μM) ( Figure 1A,B). Specifically, RA treatment led to a marked decrease in AP activity within 24 h and a moderate reduction at 48 h, with further decreases noted at higher concentrations of RA (2 μM). Concurrently, RT-qPCR analysis revealed that the expressions of differentiation-related genes such as *Gata6*, *Thbd* and *tPA* increased after 48 h of RA induction ( Figure 1C). These findings indicate that RA induces differentiation in F9 EC cells. Furthermore, both RT-qPCR and western blot analysis revealed a significant decrease in the expressions of key pluripotency transcription factors, including Nanog, Oct4 and Sox2, following RA-induced differentiation ( Figure 1D,E). Strikingly, the expression levels of PGC7 mRNA and protein paralleled the changes observed in these core pluripotency factors, suggesting a close association between PGC7 and the pluripotency status of F9 EC cells.

Our previous study demonstrated that the small molecule SC1 can maintain the pluripotency of F9 EC cells by upregulating genes associated with pluripotency, such as *Nanog* [47]. We then sought to investigate whether PGC7 accumulates during the pluripotency restoration induced by SC1. RT-qPCR revealed that *Pgc7* mRNA level was increased significantly after 48 h of treatment with SC1 (1 μM), although no significant change was detected at the 24-h time point ( Figure 1F). Furthermore, western blot analysis confirmed that Nanog expression was elevated at 48 h post-SC1 induction ( Figure 1G). In conclusion, the concurrent expression of PGC7 with the pivotal pluripotency genes *Nanog*, *Oct4* and *Sox2* during both the differentiation of F9 EC cells and their subsequent rejuvenation implies that PGC7 may serve as a positive regulator of pluripotency.

### PGC7 promotes Nanog translation

**Figure 2:**
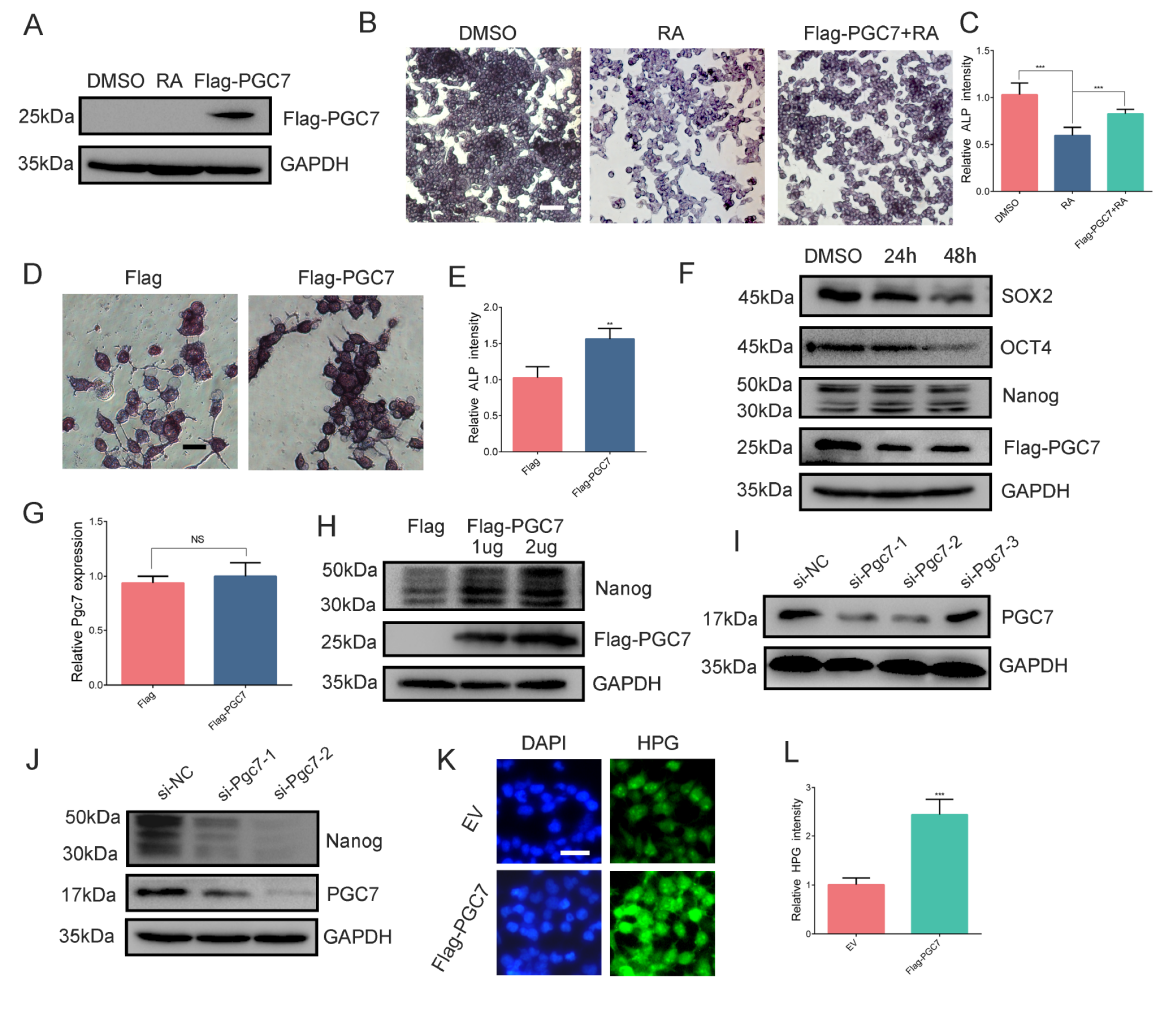
Figure 2 PGC7 overexpression promotes the translation of Nanog mRNA (A) The efficiency of exogenously overexpressed PGC7 was examined by western blot analysis. (B,C) At 6 h post-transfection with p3×Flag-CMV-10 or p3×Flag-CMV-PGC7, the supernatants of the cell cultures were removed, fresh medium was added, and the cultures were treated with an equal volume of DMSO or 1 μM RA for 48 h. AP phosphatase staining was performed to evaluate AP activity, and the intensity of AP staining was quantified via Image J software, scale bar = 80 μm. (D,E) Overexpression of exogenous PGC7 accelerated the AP activity of F9 EC cells, and the intensity of AP staining was quantified via Image J software, scale bar = 10 μm. (F) Western blot analysis of Nanog, Oct4 and Sox2 protein levels after F9 EC cells were treated with equal volumes of DMSO or 1 μM RA for 24 h or 48 h in the presence of PGC7-overexpressing cells. (G) RT-qPCR analysis of Nanog expression after PGC7 overexpression. Gapdh was used for normalization. (H) Western blot analysis of the expression levels of PGC7 and Nanog after PGC7 overexpression in F9 EC cells. (I) Detection of Pgc7 siRNA knockdown efficiency via western blot analysis in F9 EC cells. (J) Western blot analysis of the expression levels of Nanog and PGC7 after Pgc7 knockdown in F9 EC cells. (K,L) HPG fluorescence staining showing the protein synthesis activity of HEK-293T cells after PGC7 overexpression. (L) Histograms showing the HPG intensity, scale bar = 20 μm. Data are presented as the mean ± SD of three independent experiments. NS: not significant, ***P < 0.001, according to Student’s t test.

To assess the impact of PGC7 on the maintenance of pluripotency in F9 EC cells, we overexpressed Flag-PGC7 for 6 h (with the empty vector p3×Flag-CMV-10 serving as a control) and then treated the cells with RA for 36 h. Subsequent AP activity staining revealed that, compared with the empty vector control, the exogenous overexpression of PGC7 counteracted the RA-induced reduction in AP activity ( Figure 2A‒C), suggesting that PGC7 plays a direct role in maintaining pluripotency. In a follow-up experiment, we overexpressed Flag-PGC7 in F9 EC cells and assessed AP activity 48 h later. The findings confirmed that the exogenous overexpression of PGC7 similarly enhanced the pluripotency of F9 EC cells even in the absence of RA treatment ( Figure 2D,E).

To further elucidate the mechanism by which PGC7 counteracts differentiation, we performed an RA-induced differentiation assay in F9 EC cells overexpressing Flag-PGC7. The results depicted in Figure 2F revealed that the expressions of the pluripotency markers Sox2 and Oct4 decreased in a time-dependent manner following 1 μM RA treatment for 24 or 48 h, which aligns with the observations shown in Figure 1E. However, in contrast to Figure 1E, the Nanog protein levels remained stable from 24–48 h in the presence of overexpressed Flag-PGC7. This finding implies that during RA-induced differentiation, PGC7 overexpression can preserve Nanog protein expression, suggesting that the inhibitory effect of PGC7 on RA-induced differentiation is likely mediated by the maintenance of Nanog levels.

We next sought to investigate whether PGC7 regulates Nanog expression independently of RA induction. Following the overexpression of exogenous Flag-PGC7 in F9 EC cells, RT-qPCR analyses revealed that the ectopic overexpression of PGC7 did not significantly promote the transcription of *Nanog* ( Figure 2G). However, western blot analyses revealed a significant increase in Nanog protein level ( Figure 2H). These results strongly suggest that the overexpression of PGC7 enhances Nanog translation. In contrast, *Pgc7* knockdown by siRNA significantly inhibited the expression of Nanog ( Figure 2I,J). Previous studies have indicated that the PGC7-interacting proteome is predominantly involved in protein translation [42]. To validate this observation, we performed HPG staining in HEK-293T cells, which revealed that overexpression of PGC7 significantly increased overall protein translation ( Figure 2K,L). In conclusion, our findings demonstrate that PGC7 can counteract the RA-induced downregulation of pluripotency by promoting Nanog translation. The exact contribution of PGC7, whether acting independently or in collaboration with other proteins, requires further exploration.

### PGC7 promotes YBX1-S100 phosphorylation by accelerating its interaction with AKT1

**Figure 3:**
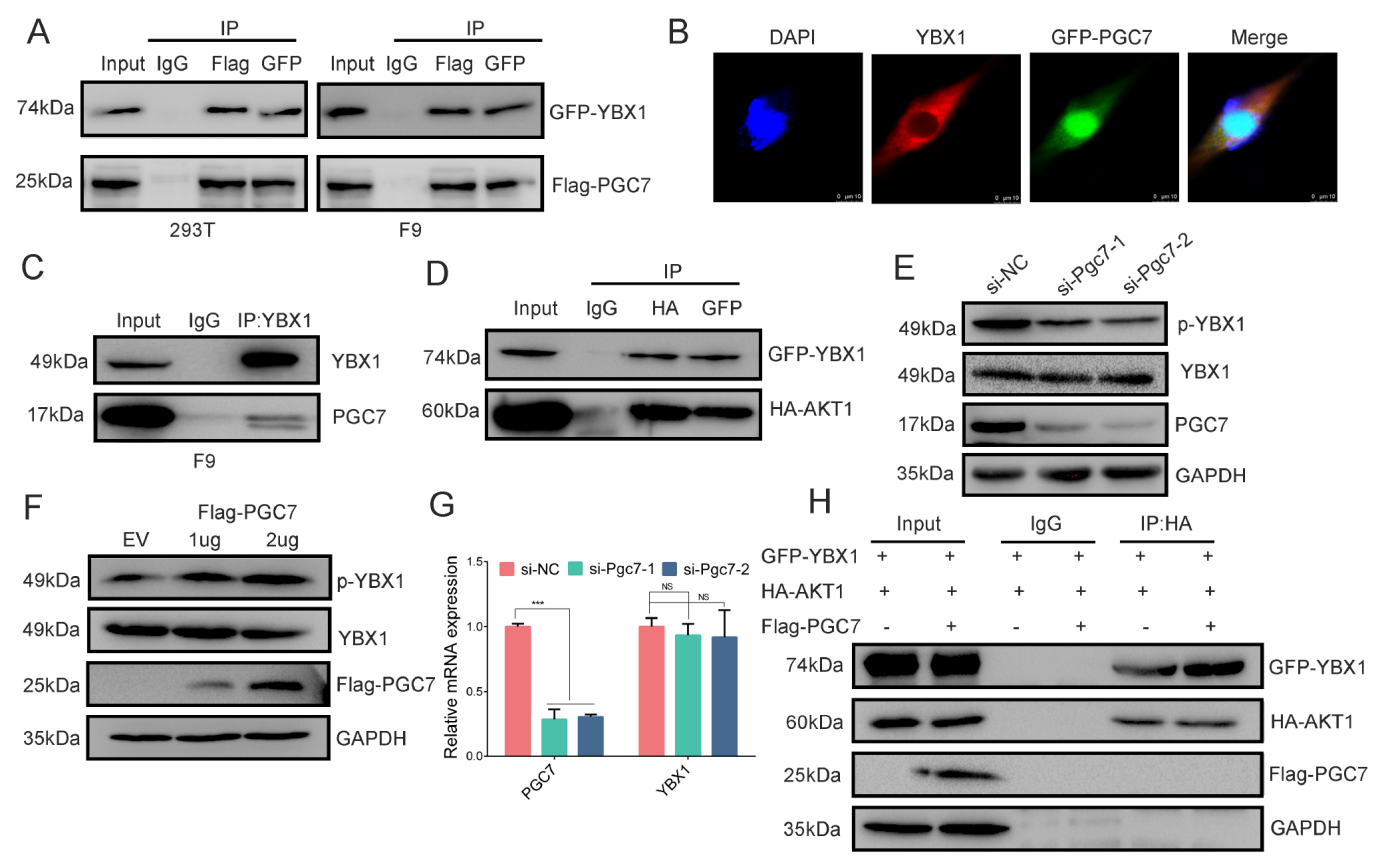
Figure 3 PGC7 promotes YBX1 phosphorylation by accelerating its interaction with AKT (A) HEK-239T cells or F9 EC cells were cotransfected with vectors expressing p3×Flag-CMV-PGC7 and pEGFP-YBX1 for 48 h, the interaction of PGC7 and YBX1 was confirmed by Co-IP. Flag-tagged antibodies were used to capture N-Flag-tagged protein PGC7 complexes, GFP-tagged antibodies were used to capture C-GFP-tagged protein YBX1 complexes, and normal mouse IgG served as a negative control. The samples were subjected to western blot analysis. (B) Confocal immunofluorescence images showing the localization of PGC7 and YBX1 in F9 EC cells. (C) F9 EC cells were used to detect the endogenous interaction between PGC7 and YBX1 when they were grown to 90% confluence. A rabbit anti-YBX1 antibody was used to capture endogenous YBX1 complexes, and rabbit IgG served as a negative control. For western blot analysis, PGC7-specific antibodies were used to detect PGC7. (D) The interaction between YBX1 and AKT1 was evaluated in HEK-293T cells via Co-IP. (E,F) Phosphorylation of YBX1 (Ser100) was analyzed by western blot analysis after Pgc7 was knocked down by siRNA (E) or when PGC7 was overexpressed (F). (G) RT-qPCR detection of Ybx1 expression after Pgc7 knockdown. Data are presented as the mean ± SD. NS: not significant, ***P < 0.001. (H) Co-IP experiments revealed that overexpression of Flag-PGC7 increases the interaction between YBX1 and AKT1 in F9 EC cells.

PGC7, although capable of binding RNA, is nonspecific [12]. These findings suggest that PGC7 may regulate Nanog translation either by directly interacting with a protein involved in translation initiation or by modulating an RNA-binding protein that plays a role in translation. Our previous study identified YBX1 as an interacting protein of PGC7, which is a crucial RNA-binding protein involved in cap-dependent translation [20]. However, the significance of this interaction, particularly with respect to the regulation of cellular pluripotency by PGC7, remains unclear. We hypothesize that PGC7 regulates Nanog translation, likely by modulating YBX1 function. To further investigate this interaction, we conducted co-immunoprecipitation (Co-IP) experiments again. GFP-YBX1 and Flag-PGC7 were co-overexpressed in HEK-293T or F9 EC cells, and GFP and Flag antibodies were used to enrich the respective proteins. The western blot analysis results demonstrated that GFP-YBX1 or Flag-PGC7 could be mutually enriched ( Figure 3A). Confocal microscopy revealed that YBX1 is strictly cytoplasmic, whereas PGC7 is distributed in both the cytoplasm and the nucleus, with interactions occurring within the cytoplasm of F9 EC cells ( Figure 3B). We subsequently utilized endogenous YBX1 antibodies to capture endogenous PGC7 in F9 EC cells, and the western blot analysis results confirmed that YBX1 can indeed capture PGC7 ( Figure 3C). Collectively, these results illustrate that the interaction between PGC7 and YBX1 is consistent. YBX1 is primarily phosphorylated by the AKT1 kinase at serine 102 (serine 100 in mice) [ 37, 48]. We then examined the interaction between YBX1 and AKT1. The Co-IP results revealed that there was indeed a clear interaction between the two proteins, which is consistent with the findings of previous studies ( Figure 3D).

PGC7 possesses a putative SAP-like domain, and the SAP domain of these proteins is known to facilitate substrate recognition and ligase activity [49]. We hypothesize that PGC7 may regulate the translation of Nanog by increasing the phosphorylation of YBX1. Downregulation of Pgc7 expression through RNA interference significantly reduced the phosphorylation of YBX1 ( Figure 3E). Conversely, 48 h after F9 EC cells were transfected with Flag-PGC7, western blot analysis revealed that the expression gradients of Flag-PGC7 corresponded precisely with the gradients of phosphorylated YBX1 ( Figure 3F). These findings suggest a direct relationship between the expression of PGC7 and the phosphorylation of YBX1. Importantly, *Pgc7* knockdown did not affect YBX1 transcription ( Figure 3G). Given that the phosphorylation of YBX1 at serine 100 is associated primarily with AKT kinase, we speculate that PGC7 may exert its regulatory influence through AKT1. In support of this theory, we observed that the overexpression of GFP-PGC7 significantly enhanced the interaction between AKT1 and YBX1 ( Figure 3H). Collectively, these results indicate that PGC7 promotes the phosphorylation of YBX1, primarily by facilitating the interaction between AKT1 and YBX1.

### PGC7 weakens the binding of YBX1 to *Nanog* mRNA through AKT-mediated phosphorylation

**Figure 4:**
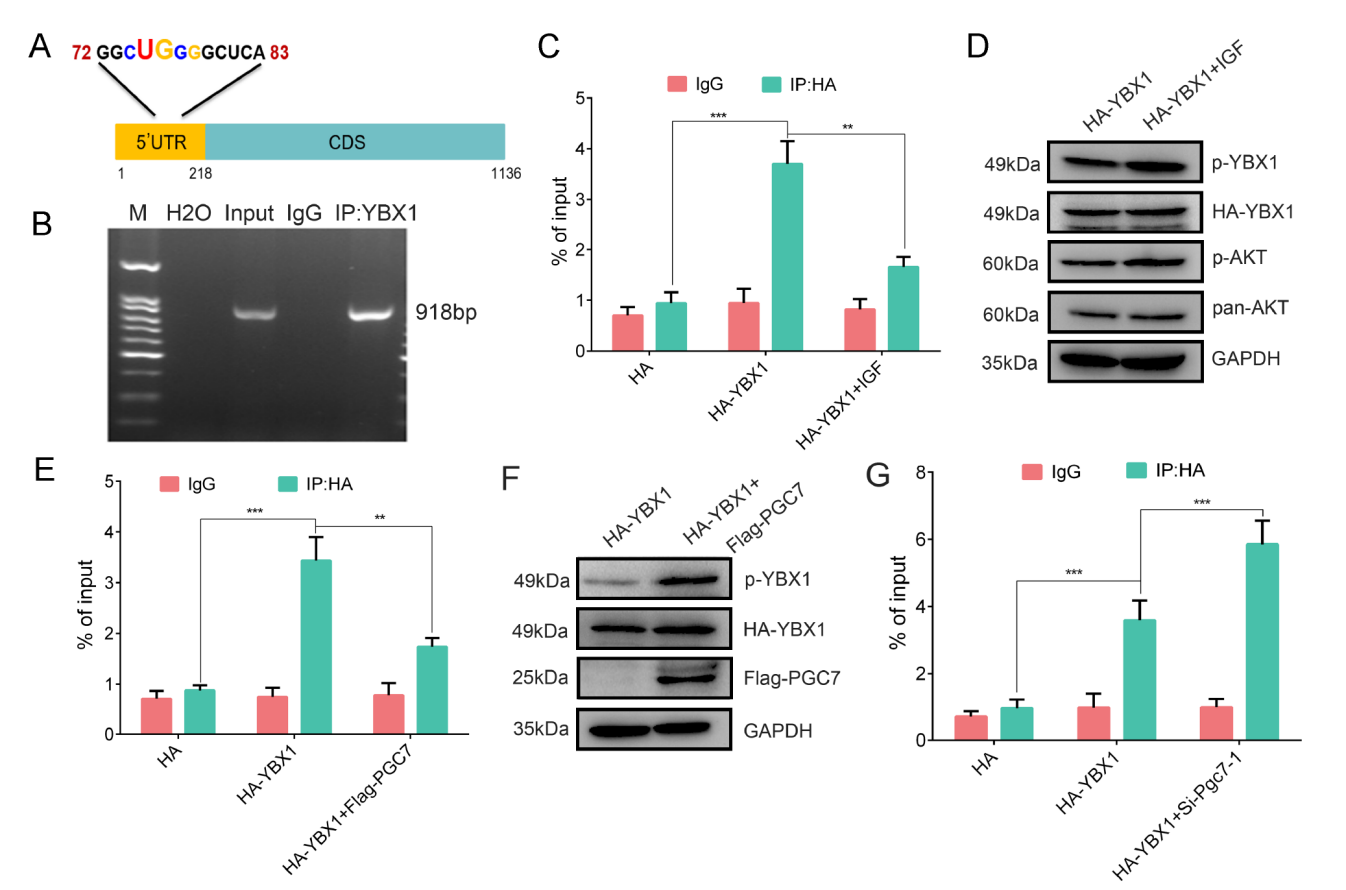
Figure 4 The binding of YBX1 to Nanog mRNA is induced by AKT (A) Schematic diagram of Nanog mRNA and a potential motif of YBX1-binding sites in the 5′UTR of Nanog mRNA. (B) The PCR product of RNA immunoprecipitation (RIP) was subjected to agarose gel electrophoresis (2%). (C) YBX1 binding to Nanog mRNA and YBX1 phosphorylation induced by IGF decreased its affinity for Nanog mRNA. RT-qPCR revealed the specific binding of YBX1 to Nanog mRNA via an RIP assay. (D) Western blot analysis of the phosphorylation of YBX1 induced by IGF. (E) Overexpression of PGC7 inhibits YBX1 binding to Nanog mRNA in F9 EC cells. RT-qPCR revealed the specific binding of YBX1 to Nanog mRNA via a RIP assay. (F) Western blot analysis of the phosphorylation of YBX1 induced by PGC7. (G) Knockdown of Pgc7 by siRNA promoted YBX1 binding to Nanog mRNA in F9 EC cells. RT-qPCR revealed the specific binding of YBX1 to Nanog mRNA via a RIP assay. Data are presented as the mean ± SD of three independent experiments. **P < 0.01, ***P < 0.001, according to Student’s t test.

AKT-mediated phosphorylation of YBX1 diminishes its affinity for the capped 5′UTR of mRNA, allowing eIF4E to displace phosphorylated YBX1 and release previously YBX1-repressed mRNAs to polysomes [27]. Recent studies have shown that YBX1 preferentially binds to sequences with complex combinations of G, C and U. Through sequence analysis, we also identified a highly probable binding site located –147 bp upstream of the transcription start site of *Nanog* mRNA, designated the UGCG box ( Figure 4A). To further confirm that YBX1 binds to *Nanog* mRNA, we conducted RNA immunoprecipitation qPCR (RIP-qPCR) assay, which confirmed the interaction ( Figure 4B). We then investigated whether the binding affinity of YBX1 for *Nanog* mRNA is modulated by its phosphorylation state, which is induced by AKT1 or PGC7 in F9 EC cells. RIP-qPCR revealed that, relative to the empty vector control, YBX1 exhibited significant binding to *Nanog* mRNA. However, following a 30-min treatment with IGF, AKT1 phosphorylated YBX1, resulting in a substantial reduction in its binding to *Nanog* mRNA ( Figure 4C,D). As depicted in Figure 4E,F, overexpression of PGC7 also led to increased phosphorylation of YBX1 and a corresponding decrease in YBX1 enrichment on *Nanog* mRNA. Conversely, *Pgc7* knockdown resulted in increased YBX1 binding to *Nanog* mRNA ( Figure 4G). These findings collectively suggest that the binding strength of YBX1 to *Nanog* mRNA is regulated by PGC7-mediated phosphorylation.

### Phosphorylation of YBX1 induced by AKT instantaneously activates Nanog translation

**Figure 5:**
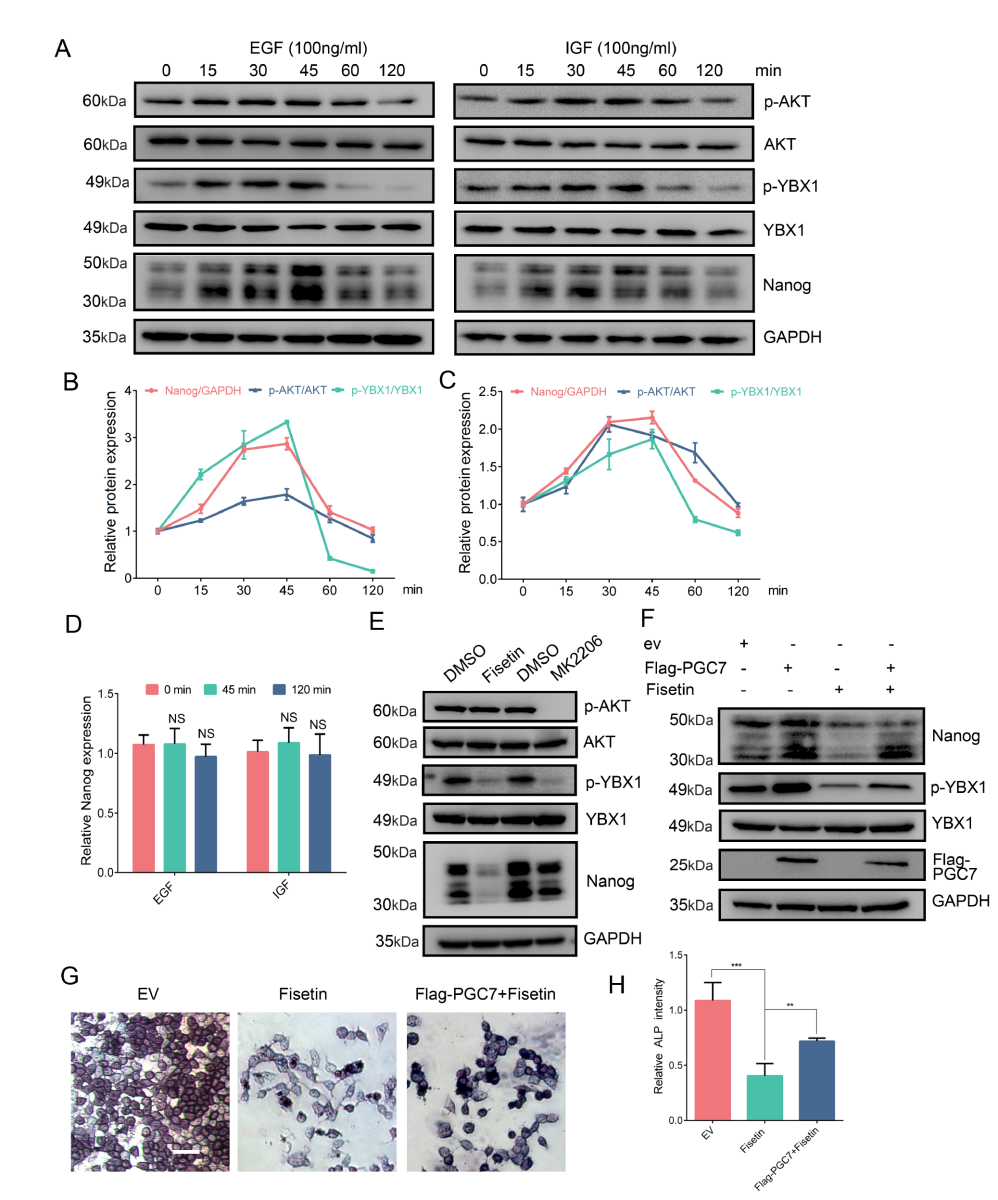
Figure 5 PGC7 promotes Nanog translation by reinforcing YBX1 phosphorylation (A) Western blot analysis of the correlation between Nanog protein expression and the phosphorylation of AKT and YBX1. F9 EC cells were starved for 12 h and stimulated with IGF or EGF (100 ng/mL each) for the indicated time. (B,C) Relative levels of protein expression from (A). (D) F9 EC cells were starved for 12 h and stimulated with IGF or EGF (100 ng/mL each) for 45 min or 120 min, and the expression of Naong mRNA was analyzed by RT-qPCR. Gapdh was used for normalization. (E) Western blot analysis was performed to detect the expression of the Nanog protein after F9 EC cells were treated with Fisetin (80 μM) or the AKT inhibitor MK2206 (5 μM) for 24 h. (F) Overexpression of PGC7 significantly rescued the Nanog expression caused by Fisetin. (G, H) Overexpression of PGC7 rescues the AP activity of F9 EC cells induced by Fisetin, and the intensity of AP staining was quantified via Image J software (H); scale bar = 30 μm. Data are presented as the mean ± SD of three independent experiments. NS: not significant, **P < 0.01, ***P < 0.001.

To elucidate the relationship between Nanog expression and the phosphorylation of AKT and YBX1, we monitored the activation (phosphorylation) of key molecules within the AKT and YBX1 pathways. As depicted in Figure 5A‒C, treatment with EGF or IGF (100 ng/mL) for 30–45 min resulted in a robust increase in AKT and YBX1 phosphorylation, which subsequently returned to control levels by approximately 120 min. Notably, Nanog expression also peaked at 30–45 min, which coincided with the phosphorylation status of AKT and YBX1. Additionally, RT-qPCR analyses revealed that the transcript level of *Nanog* did not significantly change following treatment with EGF or IGF (100 ng/mL) for 45 or 120 min ( Figure 5D).

Fisetin is a potent inhibitor of YBX1 phosphorylation [50]. We subsequently investigated the effects of inhibiting YBX1 or AKT phosphorylation on Nanog expression. Following the treatment of F9 EC cells with Fisetin or the AKT inhibitor MK2206 for 24 h, western blot analysis revealed significant inhibition of Nanog protein expression upon the inhibition of YBX1 and AKT phosphorylation ( Figure 5E). Conversely, overexpression of PGC7 was capable of rescuing the downregulation of Nanog induced by Fisetin ( Figure 5F). AP staining further demonstrated that AP activity was significantly inhibited by Fisetin but was rescued by the overexpression of PGC7 ( Figure 5G,H). These findings strongly suggest that Nanog translation is closely linked to YBX1 phosphorylation, with AKT-mediated phosphorylation of YBX1 facilitating the rapid translation of Nanog.

## Discussion

The pluripotency of ES cells is precisely regulated by core pluripotency transcription factors, including Nanog, Oct4 and Sox2. Together with Oct4 and Sox2, Nanog serves as a key transcriptional regulator of pluripotency and self-renewal [51]. Post-transcriptional regulation, particularly translational regulation, can precisely modulate the expression of Nanog in a timely manner, thereby enhancing its functional efficacy. However, studies addressing the translational regulation of Nanog are scarce.

The expression status of PGC7 has been identified as a marker of pluripotency in ES cells and germ cells. Its primary function involves modulating chromatin condensation and DNA methylation in early embryonic cells. Notably, the mechanism by which PGC7 regulates pluripotency remains unclear. We utilized F9 EC cells, which constitute a low pluripotent cell model characterized by high PGC7 expression, as the main subject of our investigation.

In this study, we noted that the expression of PGC7 closely mirrored the expression patterns of key pluripotency genes, including *Nanog*, *Sox2* and *Oct4*. During the transition out of pluripotency, PGC7 expression was downregulated ( Figure 1E) but was subsequently upregulated with SC1 treatment ( Figure 1G). Ghimire *et al*. [5] demonstrated that genes, including core pluripotency markers such as *Nanog* and *Sox2*, as well as naive pluripotency markers such as *Esrrb*, *Rex1* and *Nr0b1*, along with the PGC markers *PGC7*, *Prdm14* and *Dazl*, were downregulated in EpiSCs compared with those in LS and 2i mESCs. Compared with naive and primed ESCs, F9 EC cells exhibited low pluripotency; however, PGC7 remains closely associated with pluripotency. These findings suggest that, alongside Nanog, Sox2 and Oct4, PGC7 serves as a crucial regulator of pluripotency.

The precise role of PGC7 in regulating pluripotency, whether through interaction with other transcription factors or as an upstream regulator affecting Nanog, Sox2 and Oct4 expression, is not fully understood. Compared with cells treated with RA alone, F9 EC cells ectopically expressing PGC7 displayed increased AP activity ( Figure 2B). This observation suggests that PGC7 can mitigate the RA-induced downregulation of pluripotency. Notably, in addition to Nanog, PGC7 does not prevent the downregulation of the other two transcription factors affected by RA ( Figure 2F). Intriguingly, in the absence of RA treatment, the introduction of exogenous PGC7 in F9 EC cells did not significantly alter *Nanog* transcription; however, it significantly increased its translation ( Figure 2H). In the context of induced pluripotent stem cell (iPSC) reprogramming, exogenous PGC7 significantly enhances reprogramming fidelity, yielding a greater proportion of high-quality iPSCs by preserving the imprinting of the IG-DMR at the Dlk1-Dio3 locus through the suppression of DNMT3A enrichment [18]. Therefore, we propose that, in addition to maintaining imprinting, PGC7 may also promote the expression of Nanog, thereby enhancing reprogramming fidelity. These results indicate that PGC7 is essential for maintaining pluripotency in F9 EC cells by directly regulating the expression of Nanog.

PGC7 is a small protein with a molecular weight of 17 kDa and contains a putative SAP-like domain. Despite its potential to bind to DNA, there is no evidence in the literature suggesting that it has a transcriptional regulatory role. In somatic cells, PGC7 functions primarily to modulate chromatin condensation and facilitates epigenetic modifications. Translation regulation is mainly controlled by the interaction of trans-acting RNA-binding proteins with cis-regulatory regions in the UTRs of mRNAs, a process that occurs in the cytoplasm. Confocal microscopy revealed that PGC7 accumulates in both the nucleus and the cytoplasm ( Figure 3B). In the nucleus, it primarily interacts with chromatin to regulate condensation and DNA methylation, whereas in the cytoplasm, it may be involved in RNA splicing and protein translation. Although PGC7 can bind to RNA *in vitro*, this interaction seems to be non-specific. We previously predicted that PGC7 is an intrinsically disordered protein [19] that exhibits various biological activities through interactions with other proteins. By analyzing the interaction proteomics of PGC7, we found that PGC7 interacts with YBX1. Co-IP assays confirmed that PGC7 interacts directly with YBX1 and, more importantly, that PGC7 and YBX1 are co-localized in the cytoplasm.

YBX1 has been shown to interact with the 5′ cap complex and inhibit translation by displacing eIF4G [48]. Phosphorylation of YBX1 by AKT at S102 promotes the translation of mRNAs bound to YBX1. In contrast to its non-phosphorylated form, phosphorylated YBX1 fails to inhibit cap-dependent translation of the reporter mRNA *in vitro*. Thus, AKT may relieve the translational repression of YBX1-bound mRNAs. PGC7 promotes the phosphorylation of YBX1 at Ser100 in a dose-dependent manner by enhancing the interaction between YBX1 and AKT ( Figure 3H).

The consensus binding site in mRNAs targeted by YBX1 is known as the UG-box ( Figure 4A). Upon further analysis of the Nanog mRNA sequence, we identified a highly conserved motif in the 5′UTR that functions as a YBX1-binding site and is located between –72 bp and –83 bp upstream of the ATG codon. Considering that AKT activation leads to the phosphorylation of YBX1, it is reasonable to infer that AKT activation facilitates the timely translation of Nanog. As depicted in Figure 5A, the phosphorylation of YBX1, akin to that of AKT, peaked approximately 30–45 min after stimulation with 100 ng/mL EGF or IGF. Notably, the upregulation of Nanog expression aligns with this temporal window, although EGF or IGF does not influence Nanog transcription ( Figure 5D). These findings underscore the critical role and precise timing of AKT-mediated YBX1 phosphorylation in the translation of Nanog.

PGC7 plays a crucial role in maintaining cellular pluripotency through the intricate interplay of various signaling pathways and protein interactions. While this study focused primarily on the regulation of Nanog translation by PGC7 via the YBX1 and AKT pathways, investigating the potential interactions between PGC7 and other key signaling pathways, such as the Wnt/β-catenin or TGF-β pathways, is essential for obtaining a comprehensive understanding of the mechanisms of PGC7 in pluripotency regulation. This research not only elucidates the specific role of PGC7 in sustaining pluripotency but also holds promise for identifying novel therapeutic targets for the treatment of related diseases.

Overall, PGC7 is closely associated with the pluripotency of F9 EC cells, the correlation that is achieved by maintaining Nanog translation. By enhancing its interaction with AKT1, PGC7 interacts with YBX1 in the cytoplasm and promotes YBX1 phosphorylation at the Ser100 site. In summary, we explored the translational regulatory mechanism of PGC7 on the pluripotency factor Nanog. Furthermore, we provide a new perspective for the functional study of PGC7, highlighting its role in the regulation of pluripotency regulation.

## Supporting information

741TabS1-S5

## Supplementary Data

Supplementary data is available at *Acta Biochimica et Biophysica Sinica* online.
