# Supplementary material for: PGC7 maintains the pluripotency of F9 embryonic carcinoma cells by promoting Nanog translation: PGC7 promotes Nanog translation
Source: Acta Biochim Biophys Sin (Shanghai). 2025 Mar 11;57(9):1469–80. doi: 10.3724/abbs.2025035 (PMC12536462; doi:10.3724/abbs.2025035)
Supplement: 741TabS1-S5 [file 741TabS1-S5.docx]

**Supplementary Table S1. Primer sequence for PCR**

| Primers and restriction site | Primer sequence (5′→3′) |
| --- | --- |
| GFP-C1-YBX1-F-HindIII  GFP-C1-YBX1-R-BamHI  HA-AKT1-F-EcoRI  HA-AKT1-R-KpnI  Flag-PGC7-F-HindIII  Flag-PGC7-R-EcoRI | CCCAAGCTTCGATGAGCAGCGAGGCCGAGACCC  CGGGATCCTTACTCAGCCCCGCCCTGCTC  GGAATTCGGATGAACGACGTAGCCATTGTG  GGGGTACCTCAGATGATCCATGCGGGGC  CCCAAGCTTATGGAGGAACCATCAGAG  GGAATTCCTAATTCTTCCCGATTTTCGC |

The underlined part of the primer is the restriction site. Flag = 3×FLAG-CMV-10; HA = pCMV-HA; GFP-C1 = GFP-C1.

**Supplementary Table S2. Specific siRNA oligonucleotides for *Pgc7***

| Name | Sequence (5′→3′) |
| --- | --- |
| si-Pgc7-1  si-Pgc7-2  siRNA-NC | GCACAACGAUCCAGAUUUA  AAAGGCUCGAAGGAAAUGAGUU  UUCUCCGAACGUGUCACGUTT |

**Supplementary Table S3. Primer sequence of real-time quantitative PCR**

| Name | Primer sequence (5′→3′) |
| --- | --- |
| q-Nanog-F | CACCCACCCATGCTAGTCTT |
| q-Nanog-R | ACCCTCAAACTCCTGGTCCT |
| q-Pgc7-F | AAAGCGCCTTTCCCAAGAG |
| q-Pgc7-R | TGGCAGAAAGTGCAGAGACA |
| q-Gapdh-F | GTGTTCCTACCCCCAATGTGT |
| q-Gapdh-R | ATTGTCATACCAGGAAATGAG |
| q-Oct4-F | TCTTTCCACCAGGCCCCCGGC TC |
| q-Oct4-R | TGC GGG CGG ACA TGG GGA GAT CC |
| q-Sox2-F | GAGTGGAAACTTTTGTCCGAGA |
| q-Sox2-R | GAAGCGTGTACTTATCCTTCTTCAT |
| q-Gata6-F | TTGCTCCGGTAACAGCAGTG |
| q-Gata6-R | GTGGTCGCTTGTGTAGAAGGA |
| q-Thbd-F | CACAGGCAGTCAATGCGTG |
| q-Thbd-R | GAGCGCACTGTCATCAAATGT |
| q-tPA-F | TGACCAGGGAATACATGGGAG |
| q-tPA-R | CTGAGTGGCATTGTACCAGGC |

**Supplementary Table S4.** **Antibodies used in this study**

| Antibody | Brand | Dilution |
| --- | --- | --- |
| Rabbit Anti- PGC7 (bs-12280R) | Bioss | 1:1000 |
| Mouse anti-Flag | Thermo Scientific | 1:1000 |
| Mouse anti-HA | Sigma | 1:2000 |
| Mouse monoclonal anti-GAPDH | Proteintech | 1:1000 |
| Rabbit anti-YBX1 | CST | 1:1000 |
| Rabbit anti-phospho-Ybx1(Ser102) | CST | 1:1000 |
| Rabbit anti-Nanog | CST | 1:1000 |
| Rabbit polyclonal anti-Sox2 | Proteintech | 1:1000 |
| Rabbit polyclonal anti-Oct4 | Proteintech | 1:1000 |
| HRP-labeled goat anti-mouse IgG(H+L) | Beyotime Institute of Biotechnology | 1:2000 |
| HRP-labeled goat anti-rabbit IgG(H+L) | Beyotime Institute of Biotechnology | 1:2000 |
| IP(TM) HRP, goat anti-mouse IgG LCS | AmyJet AMJ-AB2016 | 1:2000 |
| IP(TM)) HRP, goat anti-rabbit IgG HCS | AmyJet AMJ-AB2019 | 1:2000 |
| Goat anti-rabbit IgG(H+L) highly cross-adsorbed secondary antibody, Alexa Fluor™ 546 | Invitrogen A-11035 | 1:500 |
| Goat anti-rabbit IgG (H+L) highly cross-adsorbed secondary antibody, Alexa Fluor™ 488 | Invitrogen A-11034 | 1:500 |

**Supplementary Table S5. Inhibitors used in this study**

| Inhibitor | Brand |
| --- | --- |
| MK-2206 2HCl | Abmole Bioscience; M1837 |
| Fisetin | Abmole Bioscience; M4678 |
| SC1 (Pluripotin) | MedChemExpress; HY-10579 |
| Retinoic acid (RA) | MedChemExpress; HY-14649 |
